# Mixed reality drills of indoor earthquake safety considering seismic damage of nonstructural components

**DOI:** 10.1038/s41598-023-43533-9

**Published:** 2023-09-30

**Authors:** Zhen Xu, Yajun Yang, Yian Zhu, Jingjing Fan

**Affiliations:** Research Institute of Urbanization and Urban Safety, School of Civil and Resource Engineering, University of Science and Technology Beijing, Beijing, 100083 China

**Keywords:** Natural hazards, Civil engineering

## Abstract

The damaged indoor nonstructural components in the earthquake often cause casualties. To improve the indoor earthquake safety capacity of occupants, a mixed reality (MR) drill method for indoor earthquake safety considering seismic damage of nonstructural components is proposed. First, an MR device, HoloLens, is used to capture indoor point clouds, and the indoor three-dimensional scene is reconstructed using point clouds. Subsequently, the seismic motion models of indoor components are established, so that the indoor nonstructural seismic damage scene is constructed using the physics engine and displayed using HoloLens. Finally, a guidance algorithm for a safe zone was designed for the drills. Taking a typical office as an example, an indoor earthquake safety drill was performed. The drill results show that the proposed MR method can increase the average efficiency of moving to a safe zone by 43.1%. Therefore, the outcome of this study can effectively improve the earthquake safety ability of occupants, thereby reducing casualties.

## Introduction

With advances in seismic design and construction techniques, the incidence of building structure collapses resulting from earthquakes has significantly decreased^1^. However, it is important to note that even with these improvements, nonstructural damage (e.g., fallen ceilings and overturned furniture) would still result in serious casualties^2,3^. Therefore, ensuring the safety of occupants during an earthquake is critical for reducing casualties.

Earthquake safety drills are an effective way to improve the safety capability of occupants. Current methods of earthquake safety drills mainly include practical^4–7^ and virtual drills^8–12^. Practical drills generally require participants to practice safety actions when they receive the assumptive earthquake signal, like the Great ShakeOut^13,14^ earthquake drill. While practical drills are unable to replicate the actual earthquake scenes, it is difficult for occupants to experience a sense of urgency or tension. On the other hand, virtual drills can provide highly realistic earthquake scenes, but participants are often limited to a fixed location due to the virtual equipment (e.g., VR Head-mounted display), which reduces their mobility.

Mixed reality (MR) technology^15,16^ enables participants to freely move in the real world while experiencing realistic earthquake scenes, therefore it blends the advantages of practical and virtual drills, and provides a new approach to earthquake safety drills.

Currently, Microsoft’s HoloLens^17,18^ is one of the most popular MR headset devices, which can offer some featured functions such as mobility and spatial mapping. As a result, it has been widely utilized in various MR studies, including those focused on safety drills^19–23^. For example, Sharma et al.^19^ utilized HoloLens to provide three-dimensional (3D) holographic views of building floor plans, facilitating users in finding exits during evacuations. Asgary^21^ developed a research project named Holodisaster using HoloLens for emergency management training and public safety education.

As aforementioned, nonstructural components are important factors affecting the safety of ocuppants^24^. However, the above HoloLens-based earthquake studies have focused on emergency evacuation, without considering the hazards posed by indoor nonstructural components. If the evacuation environment is added with the indoor nonstructural seismic damage, the drills can help participants better avoid injuries caused by nonstructural damaged components and improve their safety capability during earthquakes. Therefore, it is very important to fully consider the indoor nonstructural component damage in MR earthquake safety drills.

To implement such a HoloLens-based MR drill considering seismic damage of nonstructural components, the following three challenges need to be addressed.

1. How to reconstruct the indoor 3D scene with nonstructural components? HoloLens can directly scan indoor scenes and provide indoor point cloud models for seismic damage simulation of nonstructural components^25^. Since the calculation of seismic damage is independent, it is necessary to extract the detailed information of nonstructural components. However, it is difficult to obtain information on individual components from the entire point cloud. Therefore, how to accurately extract the geometric and spatial information of nonstructural components from the entire point cloud is crucial for reconstructing the indoor 3D model.
2. How to display MR seismic scenes of indoor nonstructural components? Unreasonable seismic damage scenes of nonstructural components may mislead occupants to make wrong decisions and fail to achieve the drill aims in indoor earthquake drills. Therefore, it is a challenge to create an accurate model of the nonstructural component physical processes for simulating indoor seismic damage, and to visualize the mixed-reality damage.
3. How to guide earthquake safety drills considering seismic damage of nonstructural components? Providing safety guidance can assist occupants in learning reasonable indoor earthquake safety strategies. However, occupants face difficulty in identifying dangerous zones indoors as they cannot predict the impact of complex nonstructural components during an earthquake. Moreover, in MR drills, it is crucial to consider how to provide feedback to occupants on dangerous zones. Therefore, identifying safe zones and providing feedback to occupants on dangerous zones using the constructed indoor seismic scenes is an important issue in guiding earthquake safety drills.

For challenge (1), several studies have utilized the HoloLens’ spatial mapping for scene model reconstruction^26–30^. Hübner et al.^27,28^ proposed a voxel-based approach to construct indoor models using HoloLens triangle meshes. Martin et al.^29,30^ used HoloLens localization and spatial mapping in indoor environments to reconstruct a semantic indoor scene model. However, most of the existing scene reconstruction studies focus on building interior layouts, with fewer studies on reconstructing indoor nonstructural components. Therefore, this study aims to use HoloLens to reconstruct indoor nonstructural component scenes to provide better model support for indoor earthquake safety drills.

For challenge (2), the display of seismic damage scenes for nonstructural components such as ceilings and furniture has been studied^31–34^. Hamaguchi et al.^35^ conducted shaking table tests on general full-size furniture and proposed a simple prediction method for furniture damage. Cimellaro et al.^36^ proposed a method to assess the risk of furniture tipping based on geometric parameters of furniture and seismic intensity measurements. However, these methods failed to show the damage of nonstructural components based on physical simulations. Xu et al.^37,38^ proposed a method to simulate and visualize the seismic damage of ceiling and movable furniture based on time-history analysis (THA), FEMA P-58 (i.e., a code for assessing the damage of components), and a physical engine. However, their seismic damage model for movable furniture did not analyze cases such as overturning. Therefore, based on the method of Xu et al.^37,38^, this study improved the seismic damage model of movable furniture so as to simulate and visualize the seismic damage of indoor ceilings and movable furniture.

For challenge (3), several studies on earthquake guidance and hazard identification have been conducted^19,20,39–41^. Stigall et al.^20^ used HoloLens to provide users with 3D holographic views of building floor plans and guide them to evacuate. However, most earthquake guidance methods only considered the outward evacuation, ignoring the importance of indoor shelter. Feng et al.^39,40^ proposed immersive virtual reality serious games that guide people to take refuge indoors by embedding recommended behaviors into game scenarios, however, this method is only used in virtual reality scenarios without the integration with real environment. Amin et al.^41^ employed You Look Only Once (YOLO) for target detection to identify harmful objects during an earthquake. However, this method does not predict the extent movements of the damaged components, and therefore cannot identify safe spaces. The aim of this study is to use HoloLens to identify both safe and dangerous indoor areas, and provide guidance for participants seeking safe refuge.

In this study, an MR drill method for indoor earthquake safety based on HoloLens is proposed considering seismic damage of nonstructural components. First, HoloLens is used to capture indoor point clouds, and the indoor three-dimensional scene is reconstructed by point clouds. Subsequently, the seismic motion models of indoor components are established, so that the indoor nonstructural seismic damage scene is constructed using the physics engine and displayed using HoloLens. Finally, a guidance algorithm for a safe zone was proposed based on HoloLens. Taking a typical office as an example, an indoor earthquake safety drill was performed to demonstrate the advantages of this method.

## Framework

**Figure 1:**
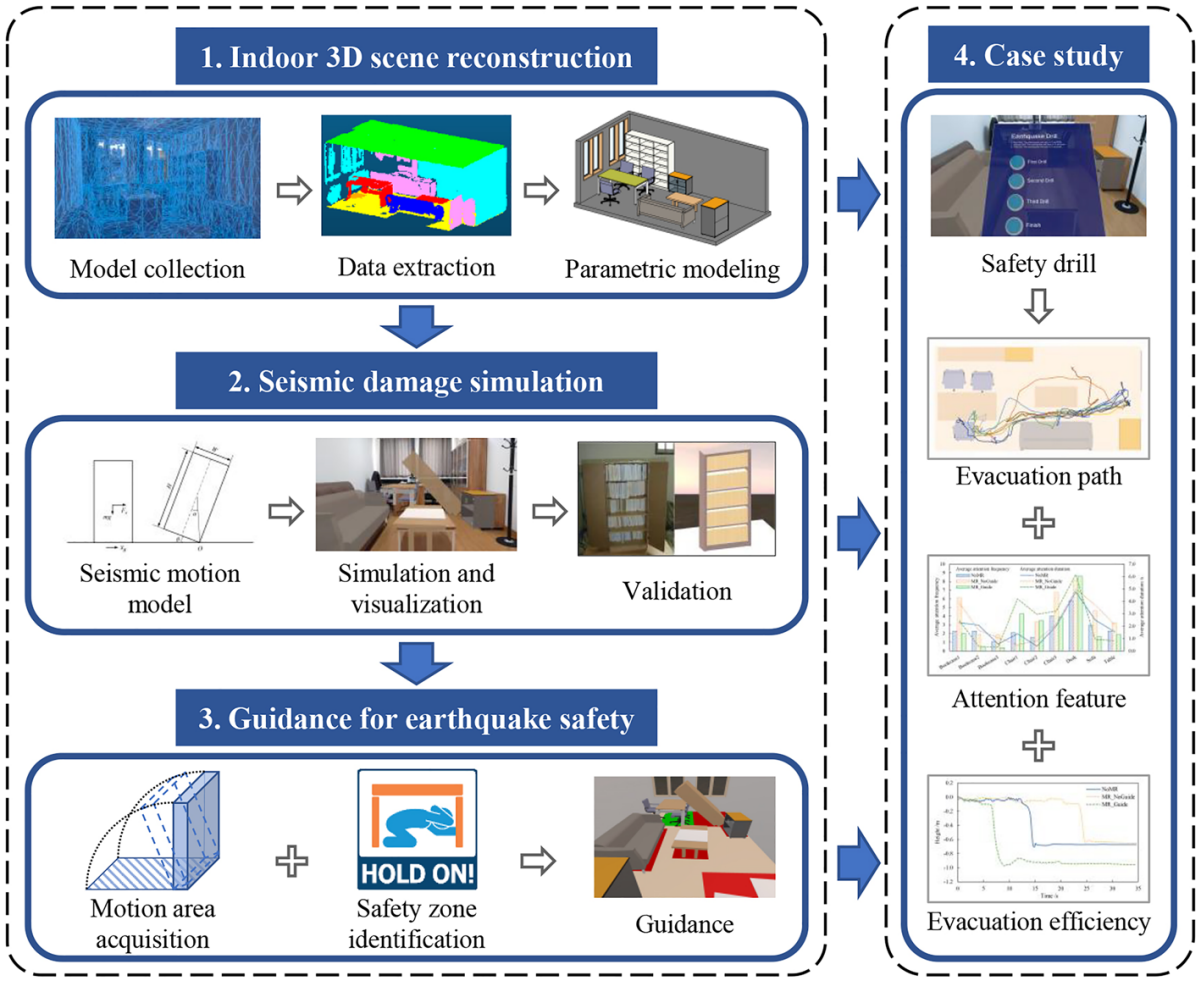
Framework of this study.

As shown in Fig. 1, the method in this study includes three parts: (1) indoor 3D scene reconstruction; (2) simulation and MR display of nonstructural component seismic damage; (3) guidance for safety drill.

1. Indoor 3D scene reconstruction. Using HoloLens spatial mapping to quickly obtain indoor 3D point clouds. Then the spatial and geometric information of indoor nonstructural components is extracted through data processing. Finally, Dynamo^42^ is used to parametrically establish the indoor building information modeling (BIM) model. Therefore, a 3D reconstruction model of a real indoor environment is provided for earthquake safety drills.
2. Simulation of nonstructural component seismic damage. Based on the BIM model, the seismic damage process for nonstructural components is calculated. Dynamic simulation in the physics engine using the THA results, so as to build a virtual scene of indoor seismic damage. The application is released on HoloLens to visualize the seismic damage scenes in MR, and the realism of seismic damage scenes is verified through a shaking table test.
3. Guidance for Earthquake Safety. The motion area of nonstructural components is obtained by calculating various parameters during the moving process. Safety zones are identified based on three discriminant conditions. Visual guidance is provided by highlighting safety, danger, and caution zones with different colors, allowing participants to quickly reach a safe location during an earthquake.

## Methods

### Ethics declarations

The study was approved by the Ethics Review Committee of the University of Science and Technology Beijing (2023-2-155). All experiments were performed in accordance with relevant guidelines and regulations in the Declaration of Helsinki. All participants were informed and informed consent was obtained to collect and publish their data and images.

### Indoor 3D scene reconstruction

The consistency between the real indoor environment and the virtual drill scene is crucial for ensuring the effectiveness of the MR earthquake safety drill. If this study chooses to directly import available 3D models of nonstructural components, it will require significant manual work such as measuring the dimensions of nonstructural components and determining their coordinates to match the real indoor environment. On the contrary, reconstructing scenes by the spatial mapping of HoloLens can easily and quickly create indoor nonstructural components and keep the consistency between the real indoor environment and the constructed scene. Therefore, this study employs indoor 3D scene reconstruction, rather than importing available 3D models.

Compared to high-cost and complex-to-operate terrestrial or mobile laser scanning (TLS/MLS) devices, HoloLens provides a low-cost and highly operable solution of spatial mapping. This study fully utilized the accessible triangle meshes generated by the spatial mapping of HoloLens 2, wherein the indoor 3D point cloud model was obtained without the need for additional tools. By adopting the 3D point cloud model for indoor 3D reconstruction, this study efficiently established a BIM model that provides the necessary information and can be converted into various model formats, such as FBX. This provides important model support for subsequent seismic damage calculations and safety drills.

**Figure 2:**
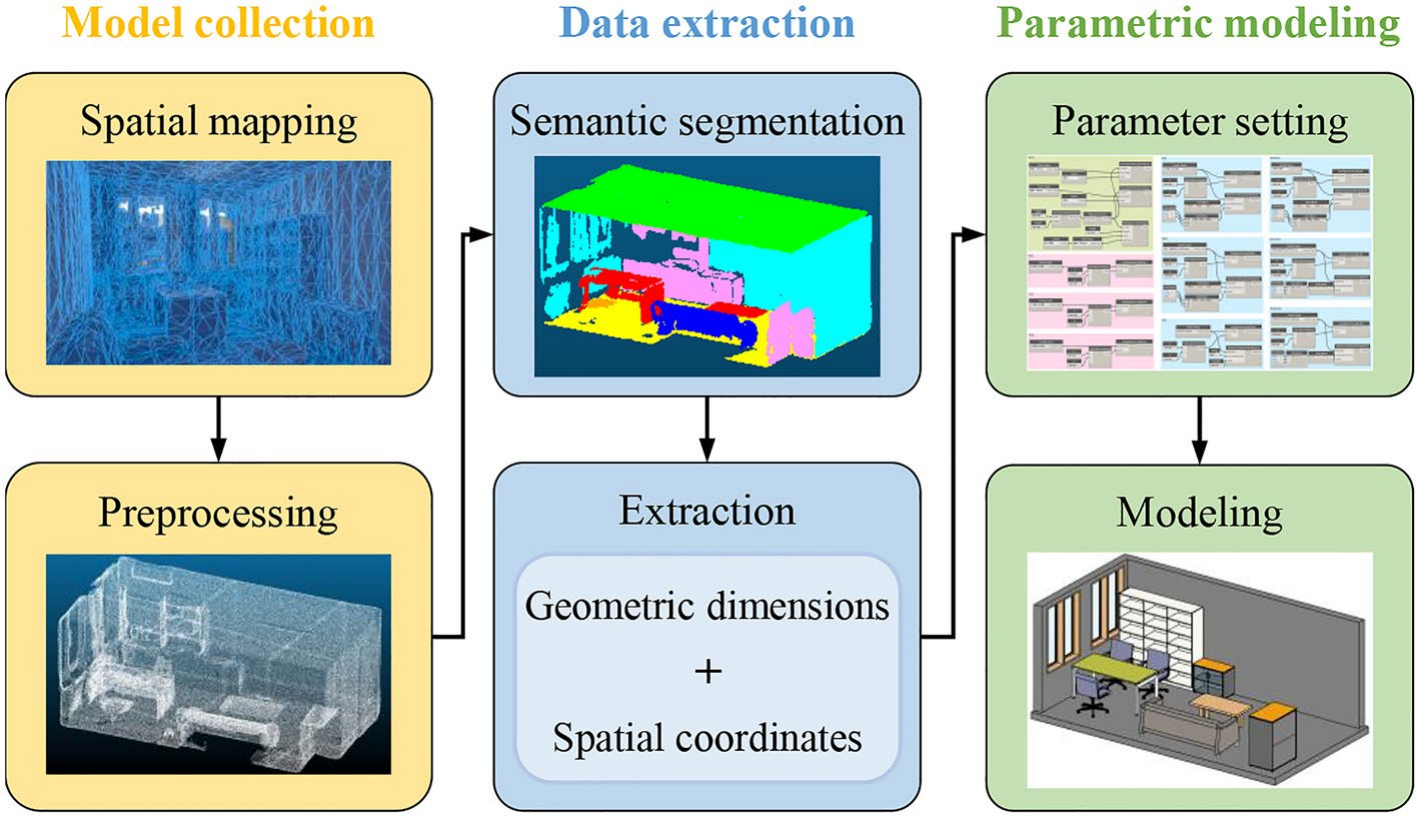
Workflow of indoor 3D scene reconstruction.

This study proposes a solution for reconstructing indoor 3D scenes using HoloLens, as shown in Fig. 2 The solution comprises three steps:

1. Model collection. The indoor scene is scanned using HoloLens 2 for generating triangle meshes and obtaining a point cloud model that can be used for subsequent calculations after preprocessing. To begin with, 3D scanning was performed with a focus on maintaining model integrity. The environment was scanned every 3 s using the spatial mapping of HoloLens. The surface grid of the collected objects could be dynamically observed, and the vacant areas were re-scanned to obtain a relatively complete model. Subsequently, the scanned model was preprocessed using the point cloud library (PCL)^43^ to obtain an optimized point cloud. Bilateral upsampling was employed to increase the resolution of the point cloud, while voxel filtering was used to remove noise points and outliers. Then, feature points were extracted to achieve registration, resulting in usable indoor point cloud data.
2. Data extraction. The point cloud model is semantically segmented to include semantic information about the nonstructural components, allowing the geometric dimensions and spatial coordinates of these components to be extracted. The geometric and spatial information of each nonstructural component was extracted. The core issue was to semantically segment the point cloud, which aims at obtaining the point cloud of each nonstructural component. Charles et al.^44^ proposed PointNet +  + , a relatively mature point cloud processing algorithm capable of achieving preferable semantic segmentation results. Therefore, the PointNet +  + algorithm was used to identify and segment the point cloud model, resulting in a point cloud model file for the nonstructural components. The 3D size of each nonstructural component was calculated, and the relative spatial position of the nonstructural components was determined by acquiring the center coordinates.
3. Parametric modeling. The BIM model is parametrically modeled to obtain a 3D reconstructed model. While commonly used BIM software such as Revit^45^ can generate BIM models that are detailed down to the component level, manually selecting walls, floors, ceilings, etc. is still necessary. To achieve automatic parametric modeling, Dynamo was selected due to its convenient visual programming environment. By importing only nonstructural component categories and the corresponding dimensional information to Dynamo, the indoor BIM model could be efficiently created for seismic damage simulation and MR display.

### Seismic damage simulation of nonstructural components

Accurate seismic damage simulation can provide effective seismic damage scenes for MR drills. This study calculated seismic damage to suspended ceilings and movable furniture, simulated the seismic damage process of nonstructural components using object motion models and physical engines, and visualized indoor MR seismic damage scenes using HoloLens.

#### Seismic motion model of nonstructural components

**Figure 3:**
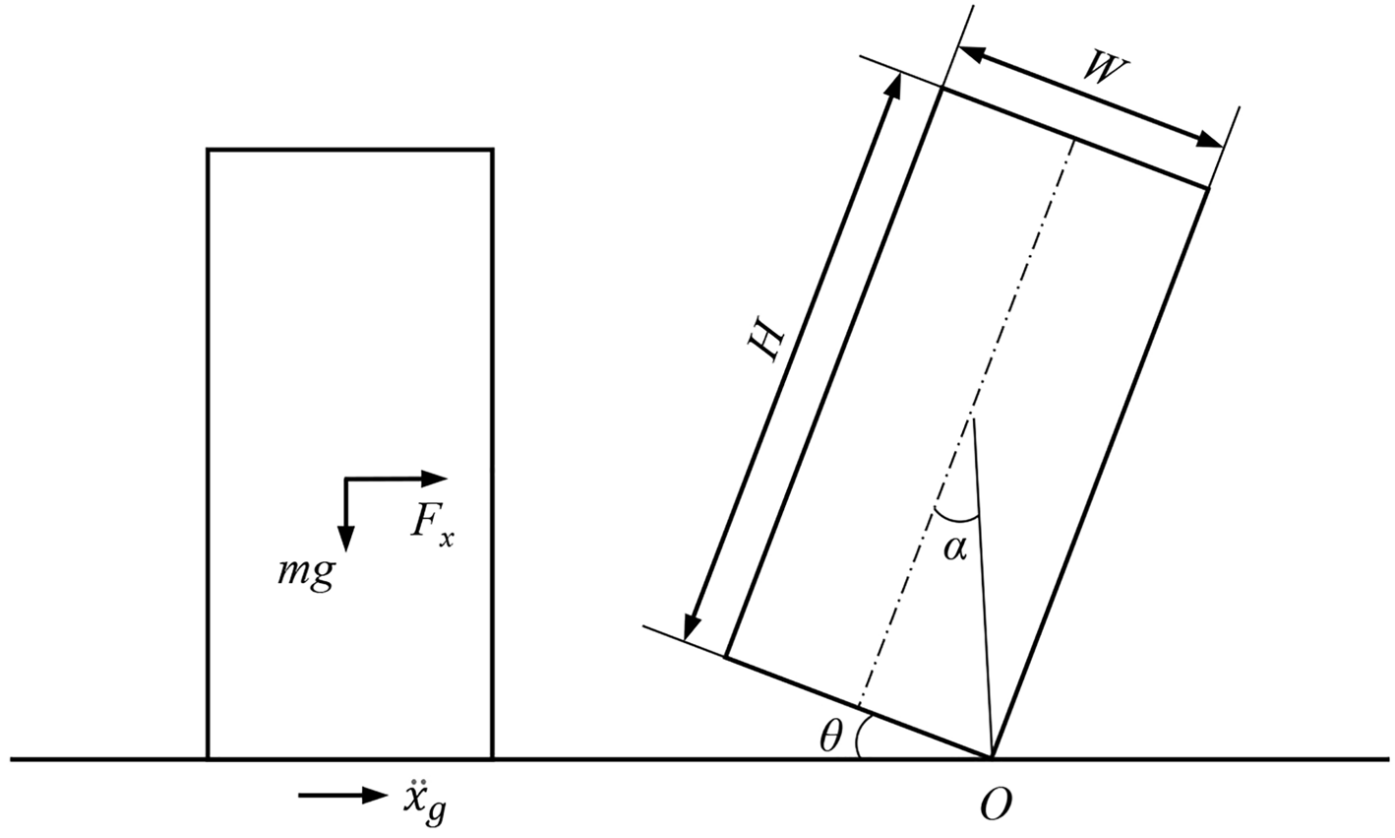
Seismic motion model of movable furniture movement.

1. Time-history analysis: According to FEMA P-58 (a national code in the U.S.)^46^, engineering demand parameters (EDPs), such as inter-story drift ratio and peak floor acceleration, are necessary for accurately calculating the seismic damage of nonstructural components. Generally, the EDPs can be calculated by the THA. This study performed the THAs of the building structures according to the reference^47^ and used the EDPs outputted by the THAs to calculate the seismic damage of nonstructural components according to FEMA P-58.
2. Seismic motion model of suspended ceiling: To calculate the seismic damage to the suspended ceiling, this study adopted the seismic motion model proposed by Xu et al.^37,38^. This model can accurately predict the falling-out state of suspended ceilings by the probability value of the FEMA P-58 vulnerability curve corresponding to the peak floor acceleration obtained through the THA. The comprehensive description of this seismic motion model of the suspended ceiling can be found in the reference^37^.
3. Seismic motion model of movable furniture: Given the various hazardous motions exhibited by movable furniture, such as rock and overturn, it is essential to obtain its motion model. The schematic of the seismic motion model is shown in Fig. 3. The movable furniture was treated as a rigid body with a mass of *m*, and an aspect ratio of $W/H$. Here *W* and *H* present the weight and the height of the movable furniture, respectively. The angle *α* is given by $\mathit{tan}\alpha =W/H$. Assuming that the center of gravity of the movable furniture was located at its center.

When the movable furniture remains stationary, the following equilibrium equations are satisfied:

1 $$\sum {F}_{x}=0, {F}_{x}=m{\ddot{x}}_{g}={F}_{y}{\mu }_{s}$$

2 $$\sum {F}_{y}=0, {F}_{y}=mg$$

3 $$\sum M=0, {I\theta =F}_{x}\cdot H/2-{F}_{y}\cdot L/2$$

where *F*_*x*_* and F*_*y*_ are the horizontal and vertical forces on the movable furniture, respectively; ${\mu }_{s}$ is the coefficient of static friction; ${\ddot{x}}_{g}$ is the horizontal acceleration of the floor under the earthquake; *M* is the moment of inertia.

When the movable furniture rocks, without considering slipping and energy loss, its motion equation can be expressed as^48,49^:

4 $$\frac{I}{mR}\ddot{\theta }+g sgn\left(\theta \right)\mathit{sin}\left(\alpha -sgn\left(\theta \right)\theta \right)=-{\ddot{x}}_{g}\mathit{cos}\left(\alpha -sgn\left(\theta \right)\theta \right), \theta \ne 0$$

where *sgn*() is the signum function.

When the movable furniture overturns, its rotation angle *θ* is always greater than the angle *α*, i.e., Eq. (5).

5 $$\theta >arctan\frac{W}{H}$$

The dynamic model subjected to earthquake (i.e., Eqs. (1)–(3)) in this study is suitable for all the furniture, regardless of the regularity of their shapes. Additionally, the calculation on furniture overturning is different for the furniture with different shapes. The calculation model on furniture overturning (i.e., Eqs. (4) and (5)) in this study is suitable for furniture with regular shapes.

#### Simulation and visualization of seismic damage

The aforementioned seismic motion models were used to simulate the seismic damage of nonstructural components, and then the simulated seismic damage scene was visualized by using HoloLens. Unity^50^, a widely-used 3D visualization platform is employed in this study for the above simulation and visualization, because it is open source and provides some good interfaces with HoloLens.

First, the indoor scene containing nonstructural components was constructed by converting the BIM models created by HoloLens into 3D models in Unity. Subsequently, the physical parameters of nonstructural components, such as friction and mass, were configured in Unity, which can be extracted from the BIM model of nonstructural components. Finally, a physics engine (i.e., PhysX) integrated in Unity was used to simulate the seismic damage of indoor nonstructural components based on the seismic motion models in this study.

For the suspended ceiling, a random function was used to select a certain number of fallings-out suspended ceilings according to the aforementioned seismic motion model proposed by Xu et al.^37,38^. The physics engine was used to activate the gravity of these falling-out suspended ceilings, and assign them instantaneous horizontal velocity of the floor, which can be obtained from the THA.

For movable furniture, according to the motion model, the inertial force and frictional force can be calculated by Eqs. (1) and (2). PhysX has embedded these equations and can be used to simulate the movement of furniture. The required floor acceleration can also be obtained from the THA.

The above constructed seismic damage scene can be visualized in HoloLens, which includes three steps:

1. Rendering configuration: The background was set to black, allowing it to be processed as transparent during rendering. The graphic quality was adjusted to ensure optimal rendering performance.
2. Spatial registration: The camera coordinates and angles were set to match the user’s line of sight and camera direction. Spatial anchors were enabled to ensure proper registration between the holographic objects and the real world.
3. Application deployment: The project generated in Unity was deployed through Visual Studio. This project will be uploaded to HoloLens device, which enables the MR visualization of the seismic damage scene in HoloLens.

#### Validation of seismic damage simulation

To validate the effectiveness of the seismic damage simulation, the existing experiment and the simulation results in this study were compared. For the suspended ceiling, the existing research conducted by Xu Zhen et al.^37,38^ has demonstrated that the simulation results were consistent with the experimental data.

**Figure 4:**
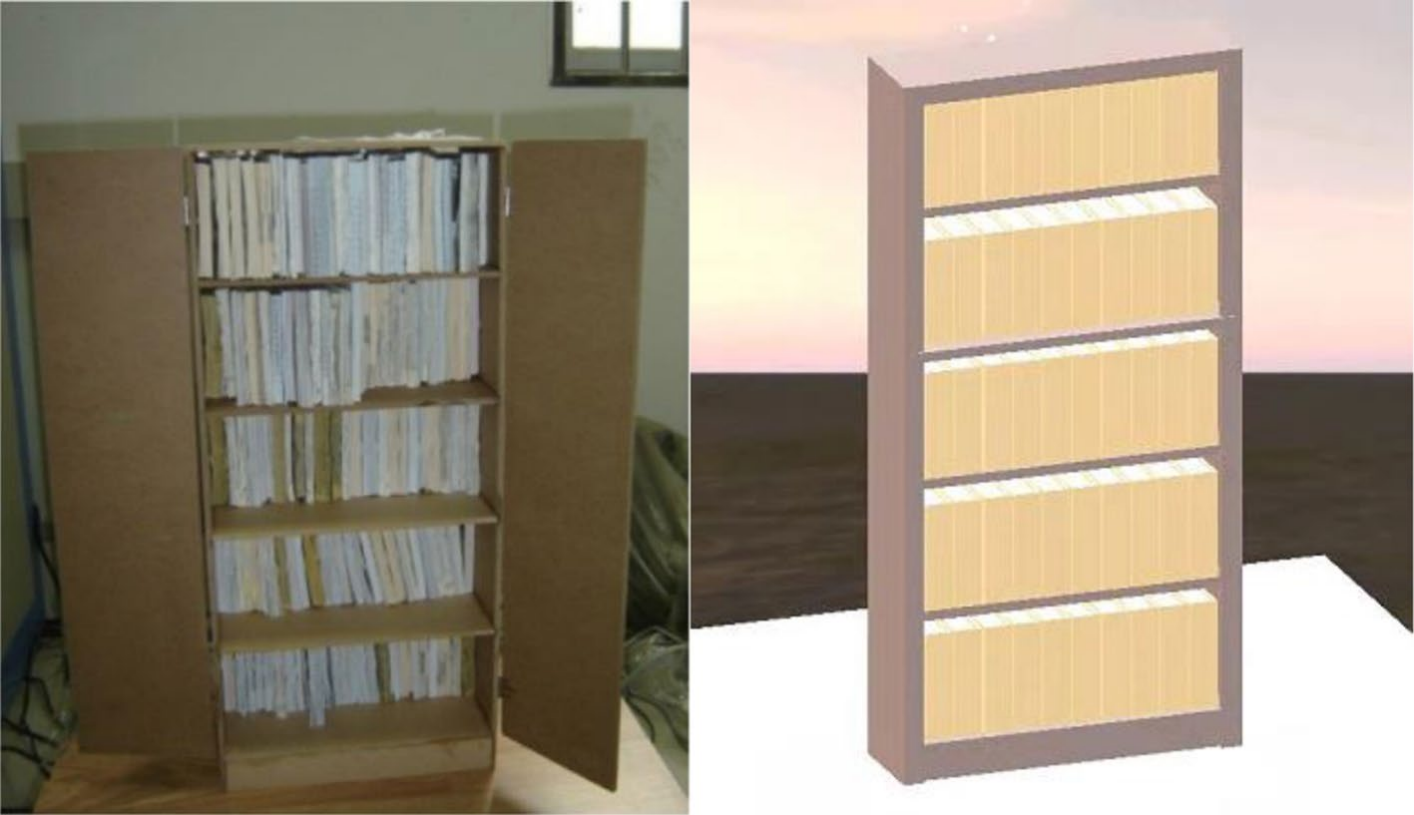
Test object (left) and simulation object (right).

**Figure 5:**
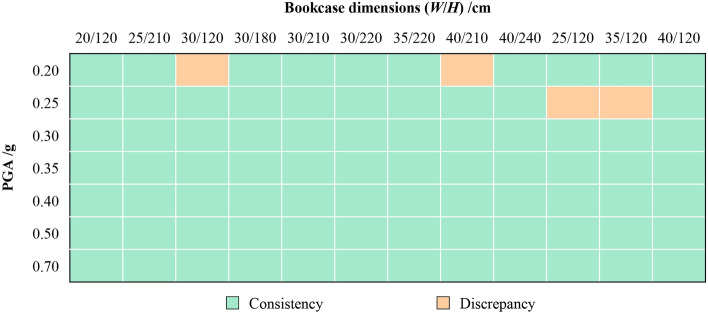
Comparison of overturning results between the test and the simulation.

In terms of movable furniture, the simulation was compared with the shaking table test of the bookcase by Luo et al.^34^ in this study, as shown in Fig. 4. In order to ensure the accuracy of simulation results, modeling was conducted in Unity using test parameters. The bookcase and books are fixed by nails and glue as a complete rigid body. A total of 12 bookcases with different dimensions were constructed according to the test. The detailed dimensions can be found in Fig. 5. The EI-Centro seismic wave was selected as the input, and the overturning states of the bookcases were recorded under 7 different peak ground accelerations (PGA) ranging from 0.2 g to 0.7 g.

Figure 5 compares the results of overturning between the test and simulation, where the green and orange colors represent the consistency and discrepancy motion states, respectively. Amongst the total 84 test cases, 80 of them had the same overturning results, resulting in a matching rate of 95.24%. This indicates that the seismic motion model can accurately simulate the motion of movable furniture during earthquakes.

### Guidance for earthquake safety

**Figure 6:**
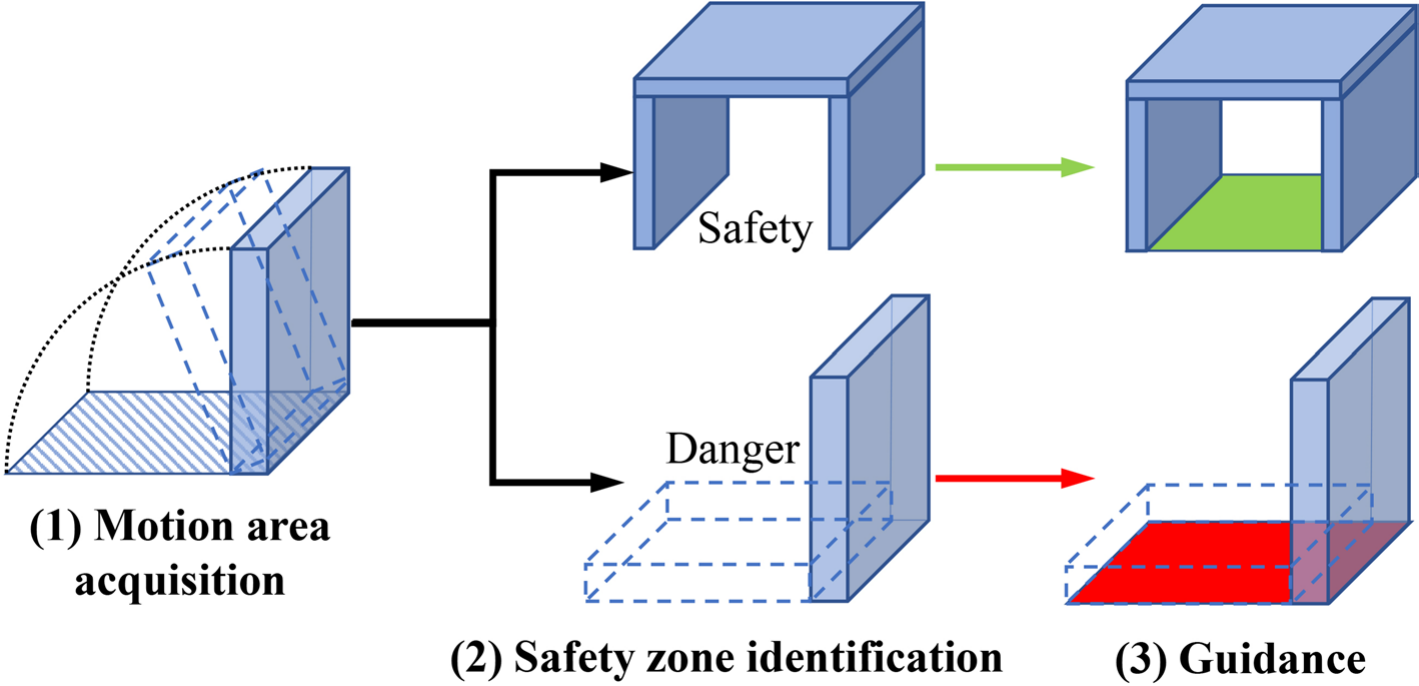
Flow chart of earthquake safety guidance.

During indoor earthquake safety drills, participants are supposed to learn proper earthquake safety strategies through guidance. According to “Drop, Cover, and Hold on” strategy^13,14^ recommended by the U.S. federal, state and local emergency management experts and other official preparedness organizations, seeking a safety zone that can cover the body of a participant is important for indoor earthquake safety drills. To help participants identify such a safety zone, this study proposed a new algorithm for guiding earthquake safety drills, which includes three parts: (1) motion area acquisition, (2) safety zone identification, and (3) guidance, as shown in Fig. 6.

#### Motion area acquisition

The motion area of the 3D models of the nonstructural components can be accurately acquired by the following four steps: (a) acquiring the center coordinates of the 3D model; (b) acquiring the model’s dimensions; (c) calculating the model’s corner point coordinates; (d) generating the motion area of the model.

**Figure 7:**
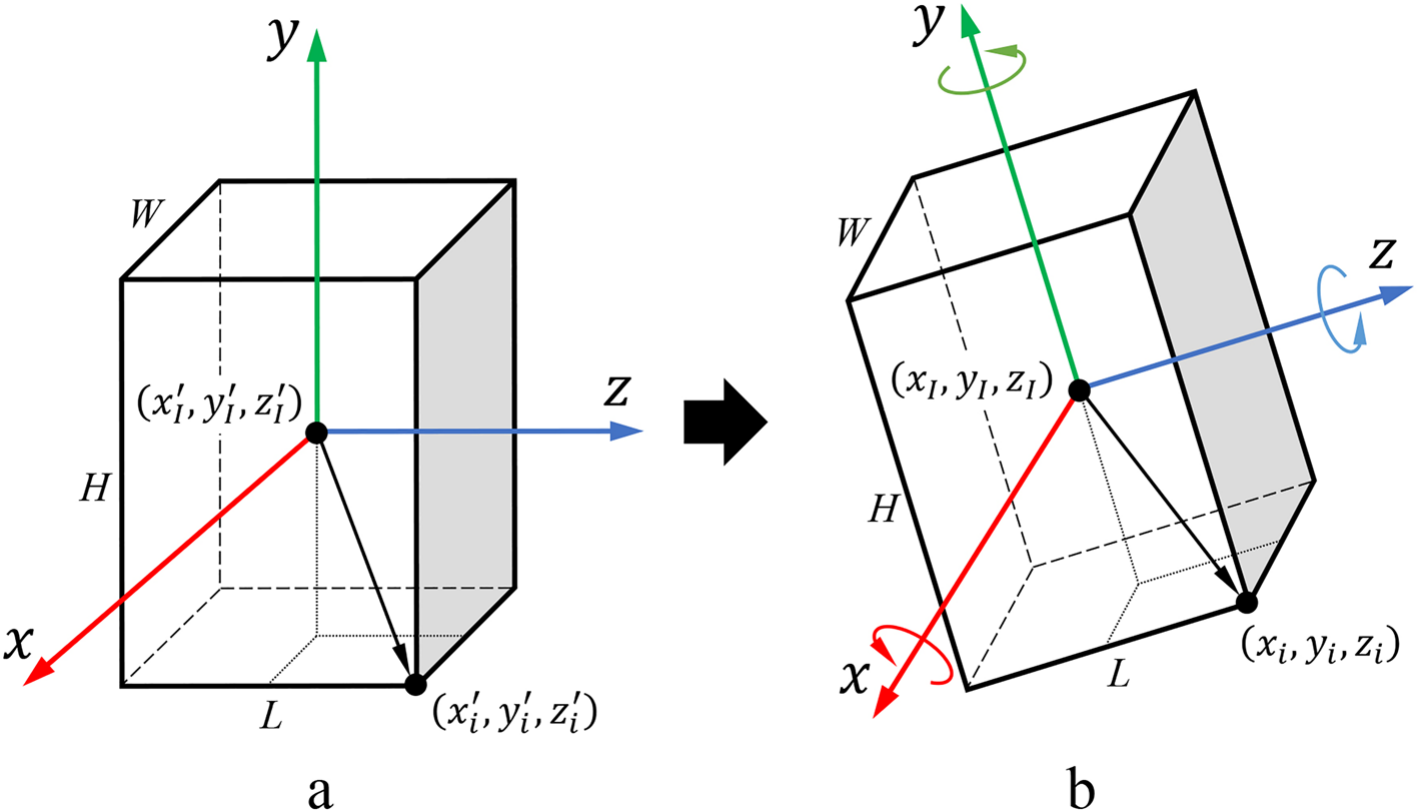
Calculation of corner point coordinates after model rotation. (**a**) Original state; (**b**) rotation state.

- Acquiring the center coordinates: In the process of modeling, exporting, and importing, it is possible that the axis of the model may not be in the center. To address this, the MeshRenderer component in Unity was used to obtain the mesh information, allowing for the calculation of the model’s center coordinates $\left({x}_{I},{y}_{I},{z}_{I}\right)$.
- Acquiring the model’s dimensions: The dimensions of the model, including the length (*L*), width (*W*), and height (*H*), were obtained using the GetComponent < MeshFilter > ().mesh.bounds.size function, from which the actual dimensions of the model can be obtained.
- Calculating the model’s corner point coordinates: To calculate the coordinates of the corner points, it is necessary to obtain the model’s rotation. However, calculations using Euler angles may encounter the problem of gimbal lock. To avoid this issue, the quaternion (i.e., Eq. (6)) was used to represent the rotation of the model.
  6 $$q=\left(w,\overrightarrow{v}\right)=\left(w,\left(x,y,z\right)\right)$$
  where *q* is a quaternion formed by combining the scalar component *w* with the vector component $\overrightarrow{v}$. According to Eq. (7), the coordinates of the corner points $\left({x}_{i},{y}_{i},{z}_{i}\right)$ were calculated using the model’s center coordinates $\left({x}_{I},{y}_{I},{z}_{I}\right)$, dimensions $\left(W,H,L\right)$, and the rotation represented by the quaternion *q* (Fig. 7).
  7 $$\left({x}_{i},{y}_{i},{z}_{i}\right)=\left({x}_{I},{y}_{I},{z}_{I}\right)+q*\left(\pm W,\pm H,\pm L\right)*1/2$$
- Generating the motion area: The corner coordinates calculated by each frame were used to generate the motion area of the model. Since the model is expected to slide, rock and overturn, the motion area should be a three-dimensional area. However, displaying a three-dimensional area in MR may obstruct the sight of participants. Therefore, the motion area was simplified into a two-dimensional plane, and the extremum values of the corner point coordinates at the plane projection were recorded to generate the plane of the motion area.

#### Safety zone identification

**Figure 8:**
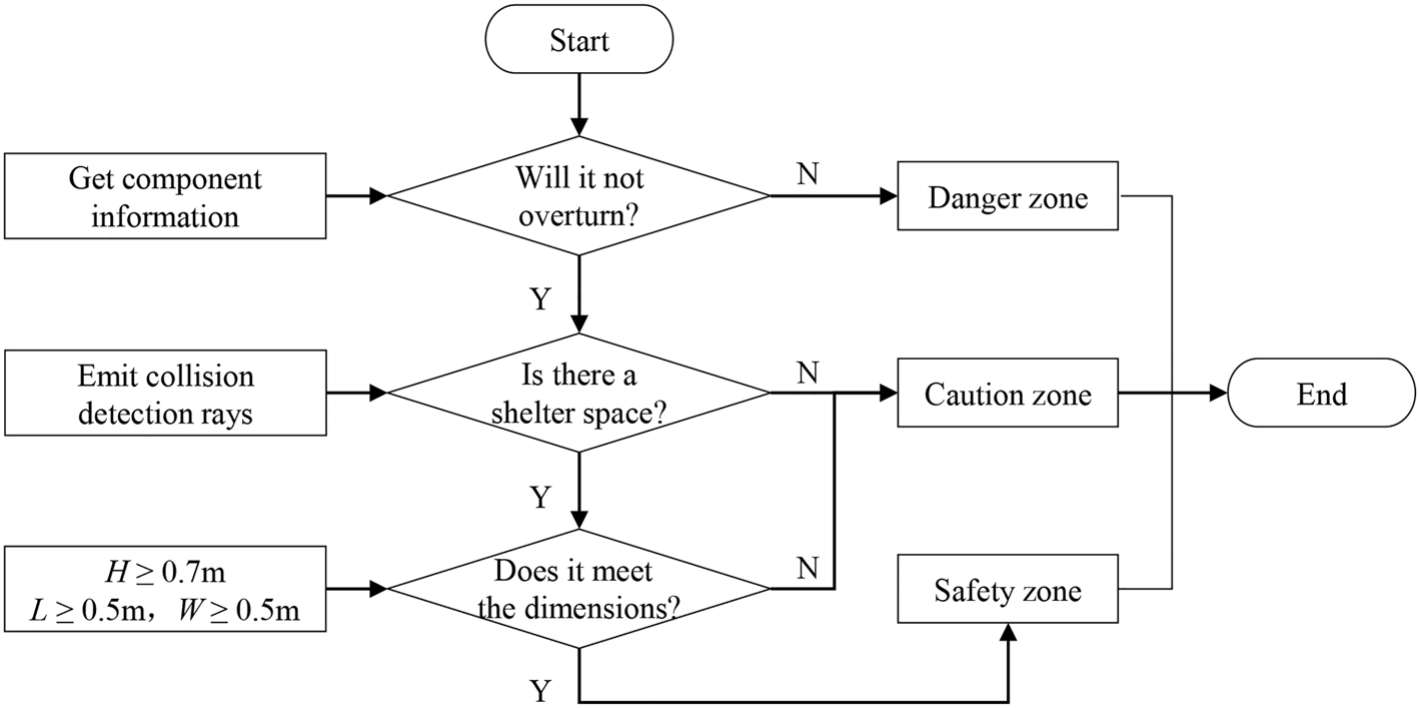
Identification workflow for the safety zone.

The identification conditions for the indoor earthquake safety zone proposed in this study were as follows (Fig. 8):

- the furniture does not overturn;
- a shelter space exists inside the furniture;
- the space’s dimensions meet the average human body.

For areas that meet all the above three conditions, they will be identified as safety zones. Areas that do not meet condition (a) will be directly identified as danger zones. Areas that do not meet either condition (b) or condition (c) will be recognized as caution zones.

For the identification condition (a), the algorithm of the motion area acquisition was used to determine the model’s rotation angle *θ*. According to the seismic motion model, when Eq. (5) is satisfied, the furniture will not overturn, satisfying the discriminant condition.

For the identification condition (b), there must be an occlusion above the shelter furniture and the interior must be hollow. Collision detection is used to confirm this. A detection ray was emitted from the ground upward and directed towards the model. If the detection ray does not collide with the bottom of the model, it is assumed that there is a shelter space in that area.

For the identification condition (c), the dimensions of the model were acquired to determine the length, width, and height of the shelter space. Based on the average size of the human body, the width of a person is assumed to be 0.5 m. The crouching height is assumed to be half of the person’s height^51^, and due to the lower posture during evasion, the height is adjusted by a reduction coefficient of 0.8 based on experimental measurements, giving a final value of 0.7 m. Therefore, if the length and width are greater than 0.5 m and the height is greater than 0.7 m, the shelter space is considered safe.

#### Guidance

**Figure 9:**
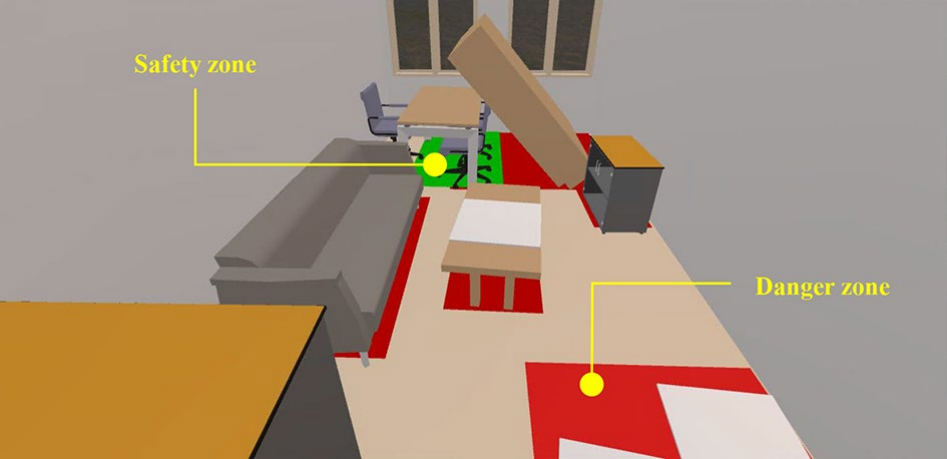
Guidance for safety zone.

Visualizing safety and danger zones can guide participants to quickly reach a shelter during an earthquake. To make the guidance clearer, the motion area was specifically highlighted, and colors were added to the different areas. Safety zones are indicated in green, while to reduce the influence on participants caused by more colors, both danger and caution zones are indicated in red. The GetComponent function was employed to obtain the rendering component of the corresponding plane, and the render.material.color function was used to set the material color of the model. By employing this visualization, earthquake safety guidance was realized, as shown in Fig. 9.

## Case study

### Drill preparations

**Figure 10:**
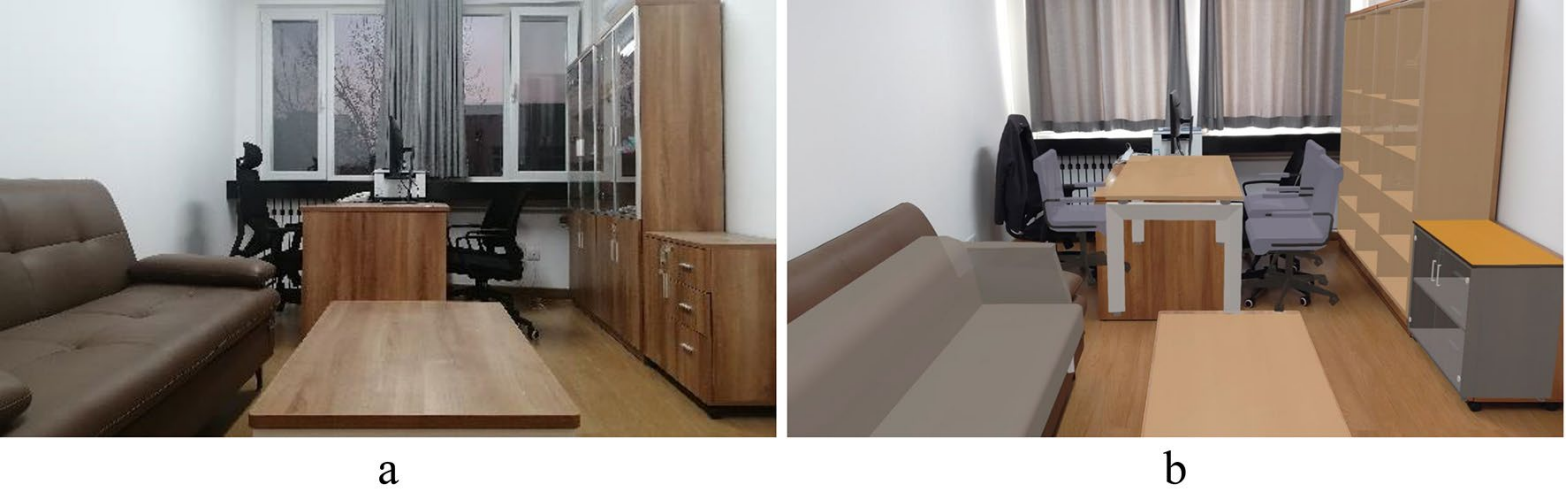
Comparison between the real environment and the MR scene. (**a**) Real environment; (**b**) MR scene.

An office in a teaching building was chosen as the drill site (Fig. 10a). The room with three bookcases and chairs, a desk, a tea table, and a sofa was scanned using a HoloLens. The acquired geometric and spatial data were then imported into Dynamo for parametric modeling, where various component types, dimension data, and spatial coordinates were assigned to generate the BIM model of the indoor 3D scene.

The indoor BIM model was imported into Unity, where corresponding properties for each component were assigned, including physical parameters, seismic damage scripts, etc. The application was then deployed to HoloLens for completing preparations before the safety drill (Fig. 10b).

### MR safety drill process

**Figure 11:**
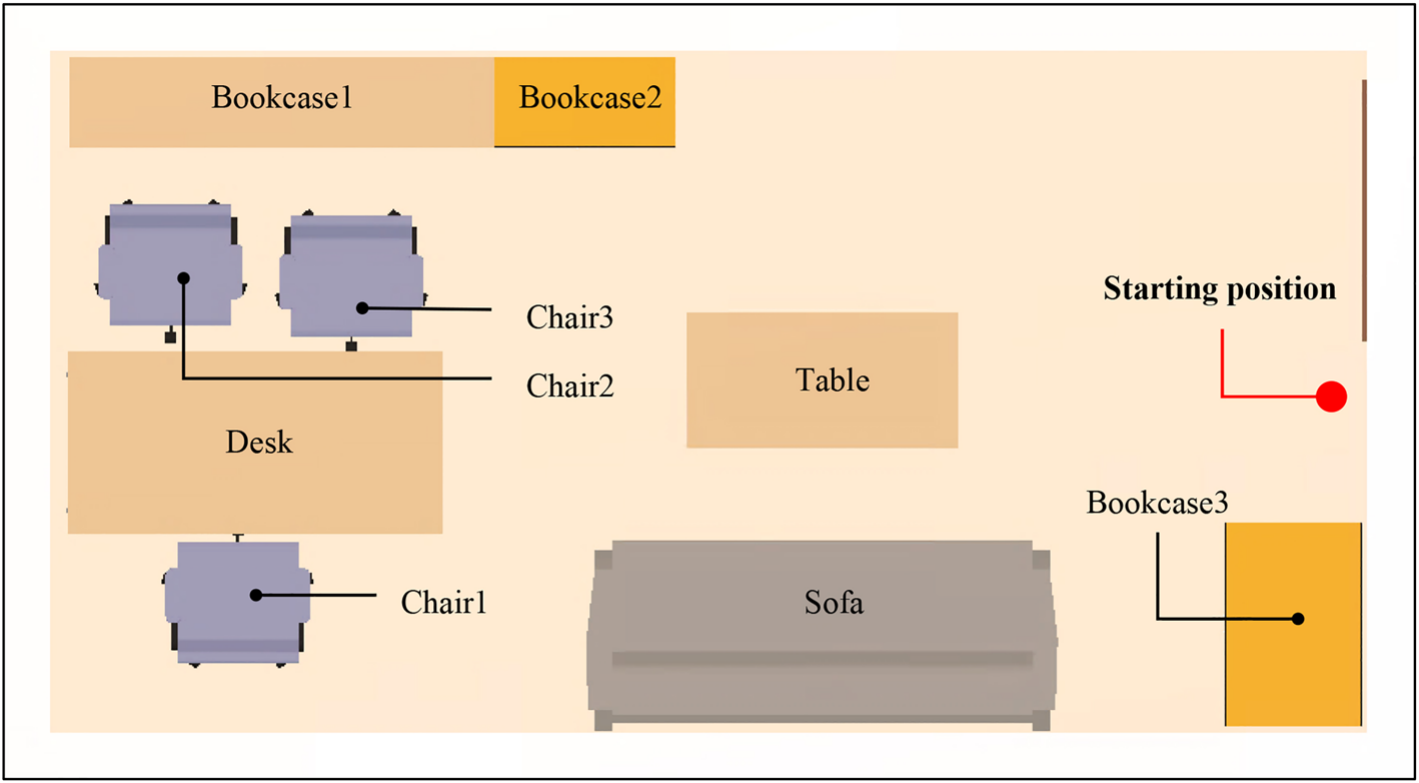
Schematic of the drill room.

**Figure 12:**
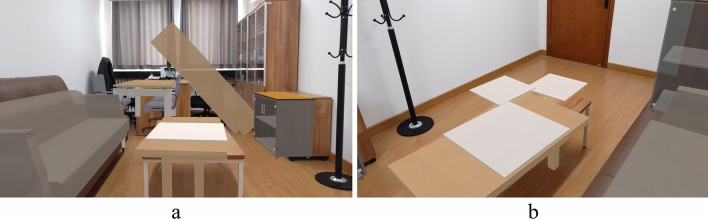
Seismic damage scenes. (**a**) View 1; (**b**) view 2.

**Figure 13:**
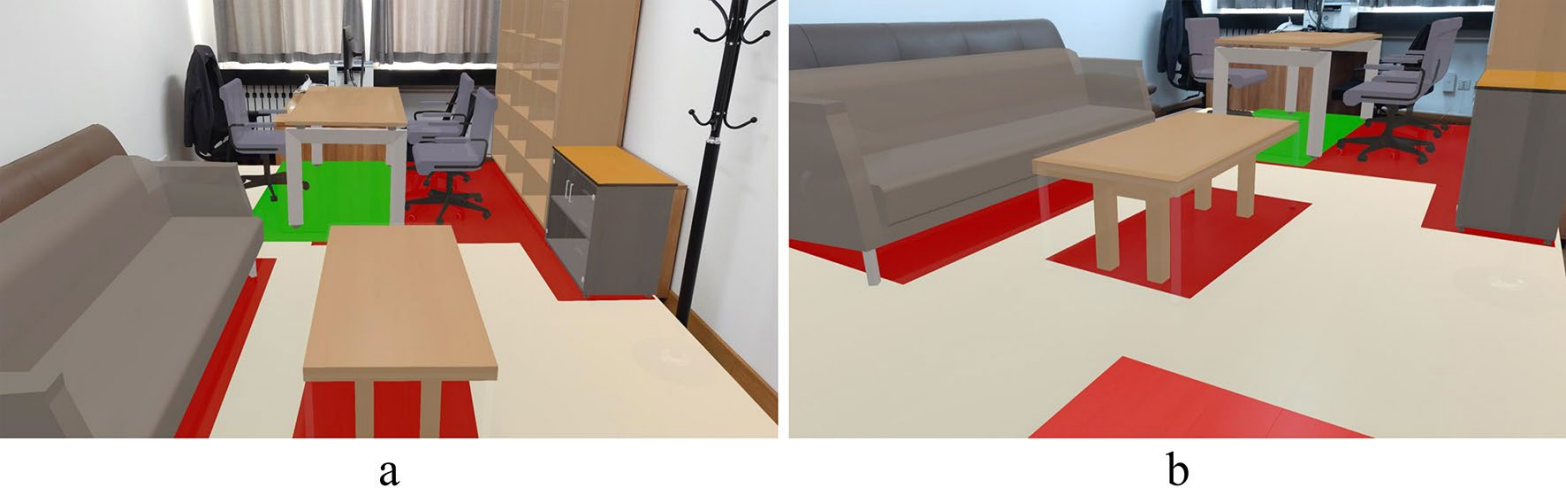
Earthquake guidance. (**a**) View 1; (**b**) view 2.

**Figure 14:**
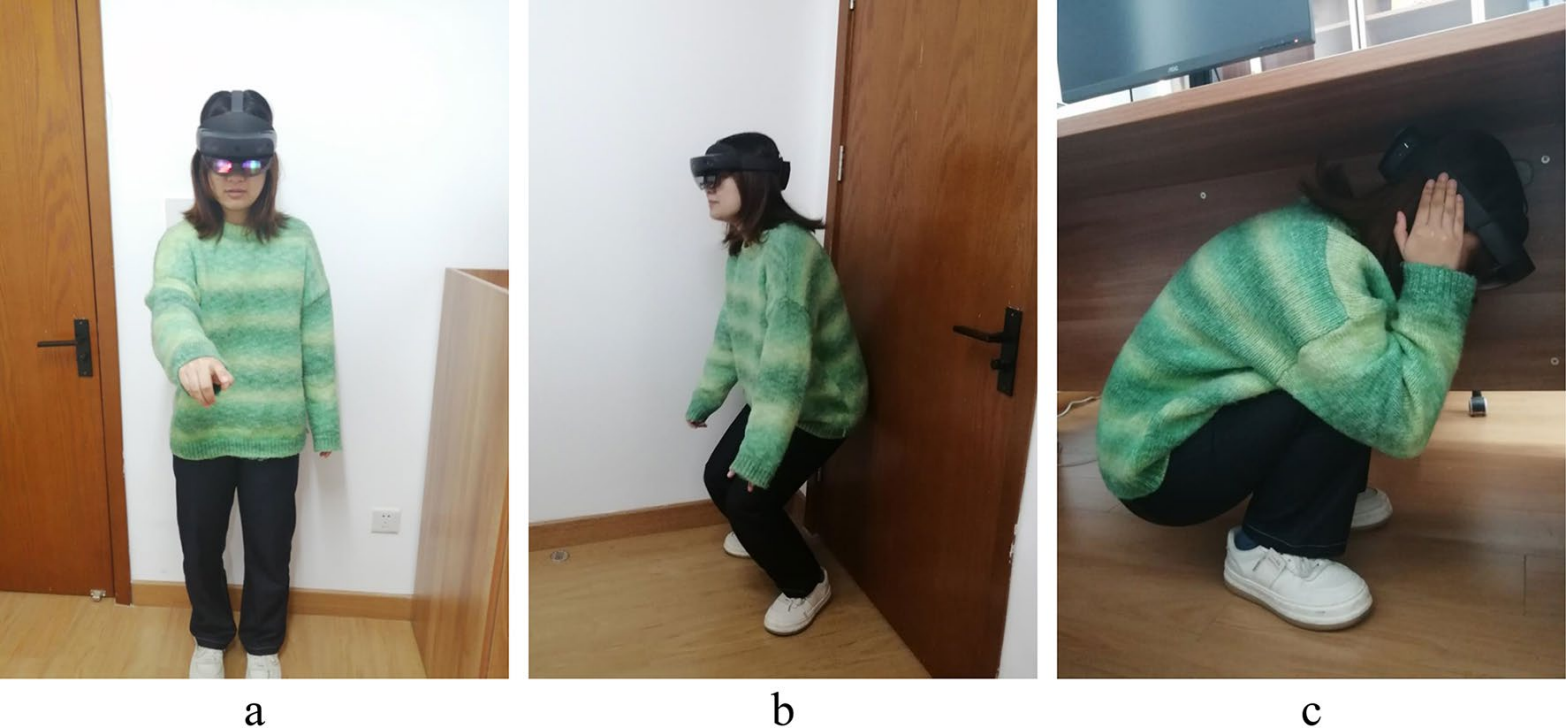
Drill process for a participant. (**a**) Drill starts; (**b**) seek shelter; (**c**) evacuation completion.

The designed MR safety drill was compared under different conditions, including with and without MR, as well as with and without guidance. The starting position of the participants is depicted in Fig. 11, which provides a global view of the indoor components, enabling participants to observe the overall seismic situation. This facilitates them to gain more information during the drill and make responsive decisions. The drills were conducted in three situations:

1. Traditional on-site earthquake safety drill in a no-seismic damage scene (NoMR): Participants evacuated based on their own experience while viewing holographic images of indoor nonstructural components, without any earthquake response or guidance.
2. MR earthquake safety drill (MR_NoGuide): Participants observed the holographic images of indoor furniture rocking and the ceilings collapsing randomly (Fig. 12), and took appropriate safety actions.
3. MR earthquake safety drill with guidance (MR_Guide): Participants were shown the holographic scene of the seismic damage and provided with earthquake safety guidance (Fig. 13). With the earthquake guidance, participants efficiently reached the safe zone. The drill process is depicted schematically in Fig. 14.

The time interval between the three drills was more than a month, ensuring participants had sufficient time to eliminate the learning influence between different drills. Additionally, a relatively ordinary office was selected as the drill scene to prevent participants from forming deep impressions of the scenes during repeated drills.

## Results and discussions

Nine volunteers participated in the indoor earthquake safety drills, and their characteristic actions were captured using HoloLens. Path tracking was utilized to record their 3D evacuation paths, while attention capture was used to record the direction of their gaze through eye tracking. The obtained data were used to analyze their safety strategies, attention characteristics, and earthquake evacuation efficiency.

### Evacuation path analysis

**Figure 15:**
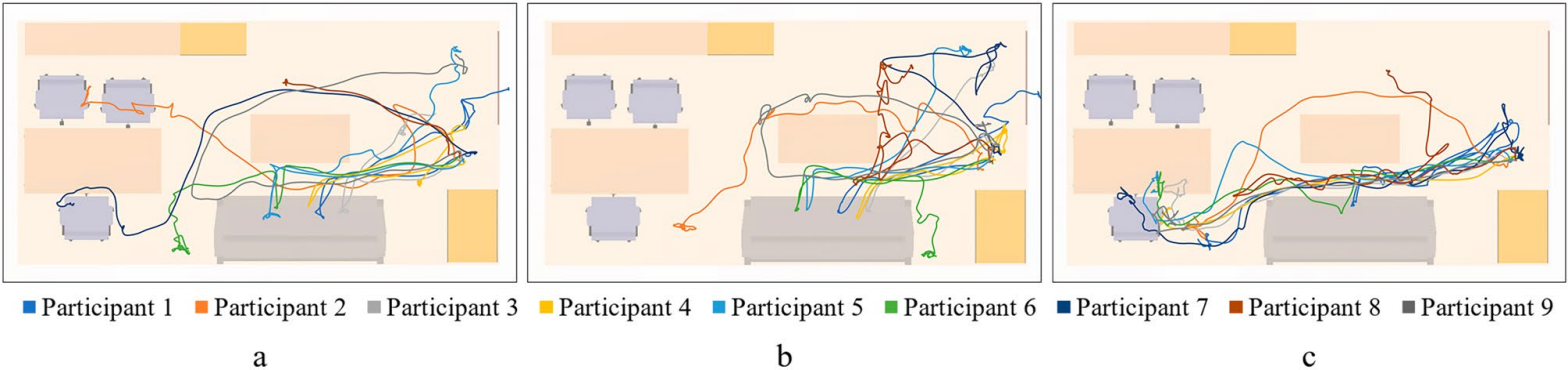
Paths of earthquake safety drills. (**a**) NoMR drill; (**b**) MR_NoGuide drill; (**c**) MR_Guide drill.

Figure 15 shows the evacuation paths of the participants, which can be analyzed to determine their final evacuation position. Additionally, the paths can reflect the evacuation features of participants. For example, if the path curves left and right, it indicates that the participants are seeking shelter; or if the path loops back and forth, it indicates the participants are exhibiting indecisive.

In the first NoMR drill, only one participant reached the safe zone, while the rest of the participants dispersed in various positions. One participant even chose to flee the room immediately when the earthquake occurred.

In the second MR_NoGuide drill, none of the participants were able to reach the safe zone, and most of them sought shelter in an open corner away from objects that rocking heavily, such as bookcases. The path of the participants was chaotic as they tried to avoid overturned furniture and falling ceilings.

In the third MR_Guide drill, eight out of nine participants successfully reached the safe zone with relatively regular evacuation paths, indicating minimal hesitation during the process. Only one participant failed to reach the safe zone. According to the interviews with participants after drills, they were afraid of the earthquake scene in MR. The participant who failed to reach the safety zone also expressed that he was too afraid to complete the drill. This further reflects the realism of the MR drill.

### Attention feature analysis

**Figure 16:**
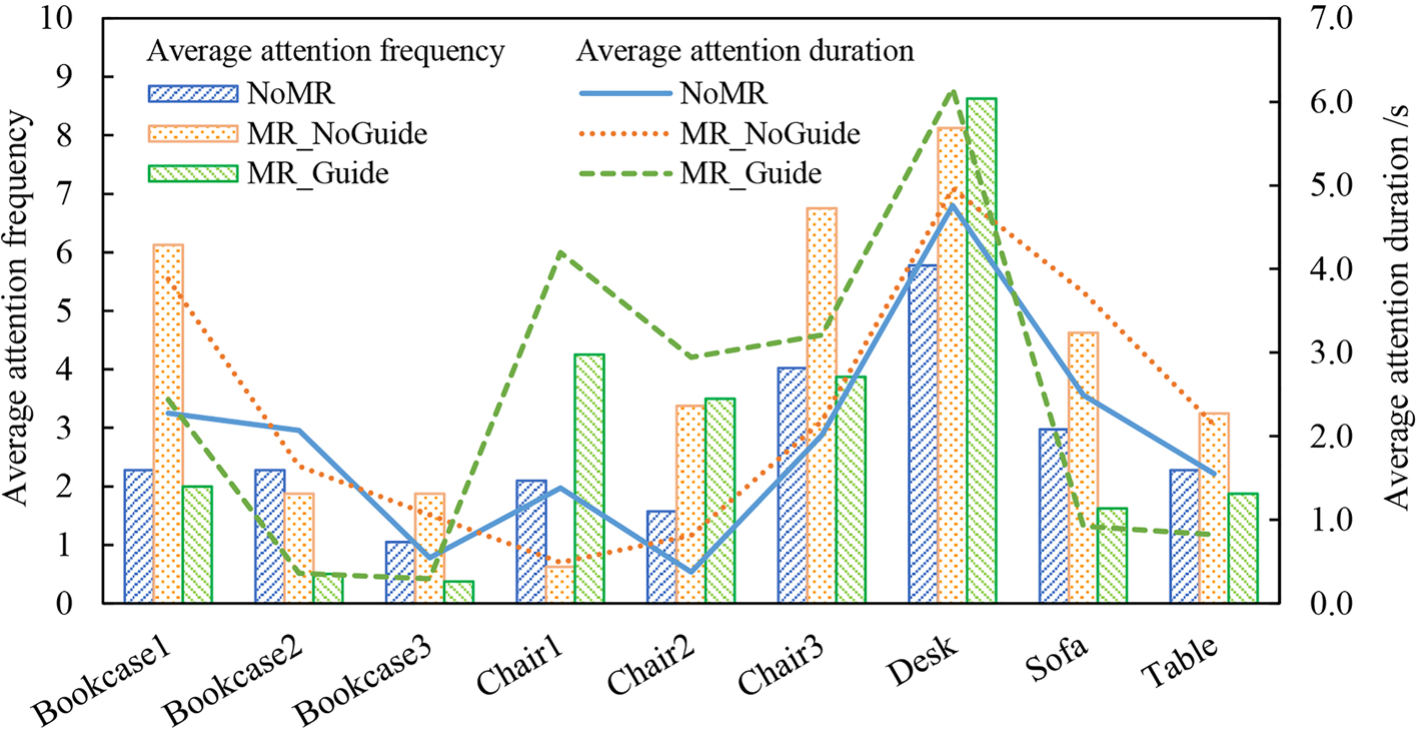
Attention statistics.

Figure 16 provides statistics on the participants’ attention to indoor components during the evacuation process, allowing for the analysis of their attention features. Notably, the participants showed a higher level of attention towards the “Desk” and “Chair 3”, likely due to their initial facing direction at the start of the drill. Therefore, it is necessary to exclude the external factors in the analysis.

In the first NoMR drill, participants’ attention to the furniture was distracted, with an average of twice.

In the second MR_NoGuide drill, after adding the MR seismic scene, participants paid more attention to the dangerous furniture that was prone to rocking. The average frequency the participants paid attention to “Bookcase 1” increased from 2.3 to 6.

In the third MR_Guide drill, with the addition of earthquake safety guidance, the average frequency participants paid attention to indoor components was significantly different. The average frequency participants paid attention to “Desk” in the safe zone reached 8.6, which is more frequent than other indoor components.

### Evacuation efficiency analysis

**Figure 17:**
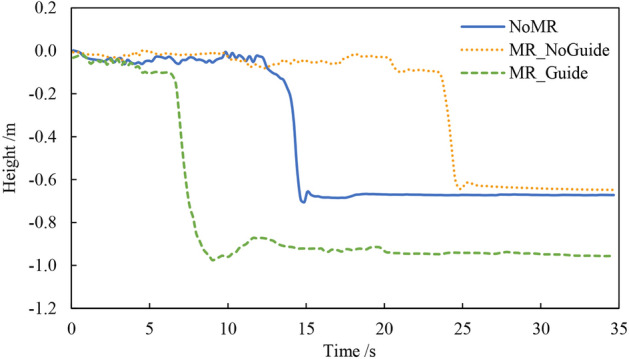
Participant’s height change processes.

In this study, evacuation efficiency is defined as the completion time taken from the start of the earthquake drill until participants complete their safety actions. By extracting the time at which the height changed, it is possible to determine the exact moment when participants completed safety actions. Figure 17 shows the height change processes of a participant in three drills, and the safety actions were completed at 14.64, 24.72, and 8.82 s, respectively.

**Figure 18:**
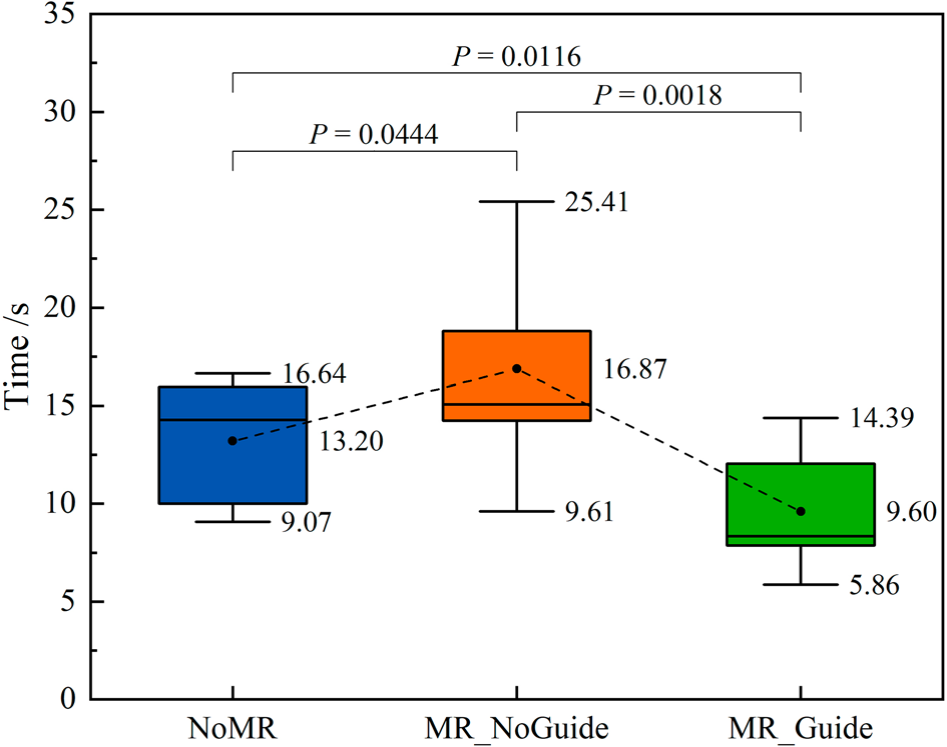
Completion time of the drills.

Figure 18 presents the completion time of the three earthquake safety drills of the nine participants. The average completion time for the NoMR, MR_NoGuide, and MR_Guide drills was 13.20, 16.87, and 9.60 s, respectively. Through the Shapiro–Wilk test, the data from the three drills all conform to a normal distribution. According to T-test, there was a significant difference between the NoMR and MR_NoGuide drills (*P* = 0.0444 < 0.05). Similarly, there are significant differences between the MR_NoGuide and MR_Guide (*P* = 0.0018 < 0.01), as well as between the NoMR and MR_Guide drills (*P* = 0.0116 < 0.05). This indicates a significant difference in the results of the three drills.

Compared to the first NoMR drill, the completion time of the MR_NoGuide drill increased by 3.25 s and the evacuation efficiency decreased by 23.9%. This is because the constructed seismic damage scene makes participants respond more realistically, leading to behaviors such as fear and hesitation. Therefore, participants need to spend more time responding and making decisions, which lengthens the evacuation process and reduces its efficiency. When compared to the MR_NoGuide drill, the completion time of the MR_Guide was reduced by 7.25 s and the corresponding evacuation efficiency increased by 43.1%, which indicates that guided MR drills greatly improved evacuation efficiency.

## Discussions

(1) Effects of MR scene on indoor earthquake safety drill.

Table 1 provides a comparison of the features between the traditional safety drill (NoMR) and the MR safety drill (MR_NoGuide).

**Table 1:** Comparisons between NoMR, MR_ NoGuide and MR_Guide.

Drill features	Indexes	NoMR	MR_NoGuide	MR_Guide
Evacuation path	Shelter position	Disperse	Disperse	Concentrated
	Path	Less chaotic	Chaotic	Relatively regular
Attention feature	Attention to dangerous objects	No	Yes	Yes
	Identify the safety zones	No	No	Yes
Evacuation efficiency	Completion time	13.60 s	16.85 s	9.60 s

In this study, participants’ evacuation responses in the MR scene are closer to the behavior in real earthquakes. They are more aware of dangerous nonstructural components, with more chaotic evacuation paths and longer completion times. As a result, MR earthquake safety drills provide participants with a more realistic experience of seismic damage scenes. Therefore, the MR drill method is instrumental in deepening participants’ impression of earthquakes and enhancing their preparedness for disasters, improving the effectiveness of earthquake safety drills.

(2) Influence of the guidance on indoor earthquake safety drill.

Table 1 also includes the comparison of the features between unguided safety drills (MR_NoGuide) and guided safety drills (MR_Guide), aiming to analyze the impact of guidance on earthquake safety drills.

In the unguided drills, participants made incorrect actions due to their lack of knowledge about indoor earthquake safety. They may hide in corners, next to bookcases, or attempt to flee the room, often ignoring the danger from the head, such as falling ceiling. By providing MR earthquake guidance, participants learned the “Drop, Cover, and Hold on” safety strategy, which will enable them to safely take cover during an earthquake. Additionally, with guidance, the evacuation efficiency of participants significantly improved, with evacuation completed in less than 10 s. Therefore, the MR guidance method can enhance earthquake evacuation efficiency and significantly improve the safety of participants.

## Conclusions

In this study, an MR method for indoor earthquake safety drills considering nonstructural component damage was proposed, and a typical office was taken as a case study. The main conclusions were drawn as follows.

1. The proposed method can reconstruct the 3D models with nonstructural components based on the point cloud data scanned by HoloLens, and accurately simulate the earthquake-induced motion process of typical movable furniture and suspended ceiling, leading to a reasonable and realistic sense of the indoor seismic damage scene. Moreover, the method can guide the safety zone for earthquake safety drills.
2. Compared to the safety drills without seismic damage scenes, the completion time of the MR drill increased by 3.25 s and the evacuation efficiency decreased by 23.9%. This is because the constructed seismic damage scene with nonstructural seismic components makes participants fear and hesitation, and thus they need to spend more time responding and making decisions. The result indicates that the MR safety drills in this study can provide realistic seismic damage scenes and make participants’ responses closer to the real earthquake.
3. The MR safety guidance can significantly improve the safety ability of participants. The drill results indicated that the evacuation efficiency increased by 43.1% due to the MR guidance.

By integrating the virtual seismic damage scene with the actual indoor environment, the MR drills enable participants to learn how to keep safe properly in the earthquake, thereby reducing casualties. However, since the furniture considered in this study is quite regular, it is possible to develop additional seismic damage models for irregular furniture, which can create more indoor seismic damage scenes in the future. Moreover, a safety scoring model can be also incorporated to provide participants with more effective feedback in the following study.

## Data Availability

The data are available from the corresponding author on reasonable request.
